# A mission‐oriented approach to cancer in Europe: a joint mission/vision 2030

**DOI:** 10.1002/1878-0261.12143

**Published:** 2017-11-09

**Authors:** Julio E. Celis, Dainius Pavalkis

**Affiliations:** ^1^ Danish Cancer Society Research Centre Copenhagen Denmark; ^2^ Department of Surgery Lithuanian University of Health Sciences Kaunas Lithuania

## Abstract

By combining innovative prevention and treatment strategies in a sustainable state‐of‐the‐art virtual European cancer centre/infrastructure, it will be possible by 2030 to achieve a long‐term survival of 3 out of 4 cancer patients in countries with well‐developed healthcare systems. Furthermore, the proposed concerted actions will pave the way to handling the economic and social inequalities in countries with less developed systems. These efforts will also ensure that in the long‐run, science‐driven and social innovations reach patients across the healthcare systems in Europe.

## 1 The challenge

Today, cancer is one of the major health problems affecting our society, a situation that is set to deteriorate globally as the population grows and ages. According to the International Agency for Research on Cancer (IARC), in 2012 there were 1.28 million cancer deaths in the European Union (EU‐28), a number that is projected to increase by 30% to 1.67 million in 2030. At the same time, the number of patients newly diagnosed with cancer in the EU is expected to rise to 3.3 million from 2.6 in 2012 (Ferlay *et al*., 2013).

As a result of more effective treatments and growing number of lines of treatments, cancer is gradually becoming a chronic disease, and the prevalence will rise considerably in countries where life expectancy is already high, as is the case in the EU. Today, cancer places a substantial extra demand on the healthcare systems due to the required surveillance and continuing treatment of both the disease and the observed side effects, and as indicated by the European Academy of Cancer Sciences (EACS), ‘the formidable healthcare problem will be difficult to control unless cancer research improves disease outcome or prevents disease. Prevention, screening, diagnosis, and improved treatment and care are major strategies that may also reduce mortality rates. Furthermore, given the large number of cancer patients and survivors, focusing on their quality‐of‐life is fundamental’ (http://www.europeancanceracademy.eu/user_uploads/files/EACS%20Mission%20and%20Vision%20FINAL.pdf).

Even though much hope has emerged from recent advances in our understanding of the molecular mechanisms underlying the disease(s), the pathways through which discoveries translate into therapeutics and diagnostics that benefit patients are complex and challenging to navigate, and as a result, the process is slow, lengthy and in most cases, inefficient (Celis and Pavalkis, 2017). Translational cancer research has the patient at the centre and requires multidisciplinarity, that is collaboration between basic researchers having different expertise, clinical oncologists, pathologists, radiation oncologists, surgeons, epidemiologists, patients, universities, industry and SMEs, healthcare professionals, regulatory bodies and funders. Also, it requires scores of patients, sharing of research and clinical data and access to infrastructures and new technologies, all of which must be cross‐compatible, responsive to the needs of research and of sufficiently large scale to enable studies of statistical significance ranging from comparative efficiency to outcome research (Celis and Pavalkis, 2017).

Currently, there are many barriers to translational research that hinder the process, particularly in Europe (Celis and Pavalkis, 2017); these include (i) the intrinsic complexity of cancer, both histological and molecular; with a large number of different cancer subgroups, new types of collaborations are mandatory to reach critical mass, (ii) inadequate research coordination at national, regional and EU level, (iii) lack of appropriate funding mechanisms to provide sustainability, (iv) regulatory, ethical, educational and workforce issues, (v) lack of venture capital, (vi) modest support for high‐end technology platforms, (vii) lack of models to reward team efforts, (viii) insufficient collaboration between DG R&I and DG SANTE and (ix) lack of harmonization of EU and Member States’ priorities and policies. The situation is not made easier by the fact that health is not a competence of the EU, while research is. Additionally, research and health ministers seldom collaborate, a fact that is not optimum for fighting a complex disease(s) such as cancer.

The breadth and scope of these challenges have called for a change in the cultural attitude towards cancer research in Europe, especially in today's era of personalized/precision cancer medicine (Celis and Pavalkis, 2017; Mendelsohn *et al*., 2012, 2015). A shift from regional/national efforts into continent‐wide collaborations and a concerted effort involving all stakeholders is deemed critical to accelerating the pathway from laboratory discoveries to diagnostics and treatments that meet the needs of patients and that healthcare systems can afford. Moreover, decision‐makers need to recognize that sustaining health research, in the long term, will bring significant benefits for European citizens and the economy, as health is wealth! (http://www.euro.who.int/__data/assets/pdf_file/0008/88613/E91438.pdf?ua=1). There is evidence that research has a delayed, but real impact on patients.

**Figure 1:**
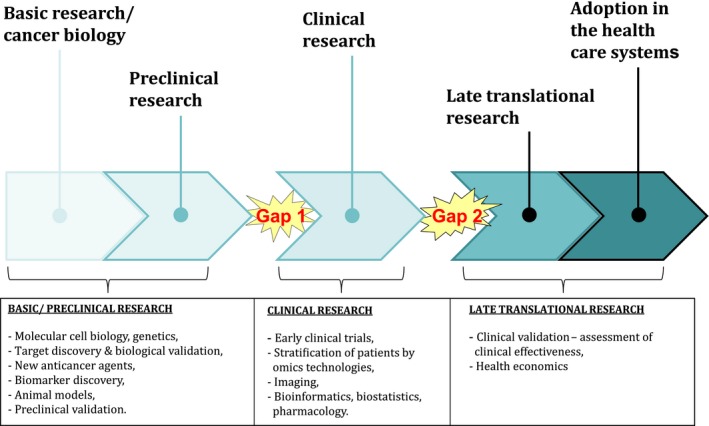
Translational Cancer Research – a coherent research continuum. Kindly provided by U. Ringborg, Karolinska Institutet.

Ideally, the creation in the long run of a healthy, sustainable virtual European Cancer Institute (ECI) with significant critical mass could provide a platform for pan‐European research of excellence aimed at fighting cancer in partnership by sharing the highest standard of practices and big data among all participating countries and centres across Europe and beyond. In particular, engaging Comprehensive Cancer Centres (CCCs) is crucial, as these institutions link treatment and prevention with research and education and connect research with the healthcare systems, thus allowing early and late translational research, the latter designed to determine clinical utility and cost‐effectiveness (Fig. 1) (Celis and Pavalkis, 2017). An important mission of the CCC is to quality assure cancer care (covering all diagnostic and treatment modalities such a surgery, radiation therapy and medical oncology, including rehabilitation, psychosocial oncology, supportive care and palliative oncology) and disseminate innovations in a defined outreach area.

**Figure 2:**
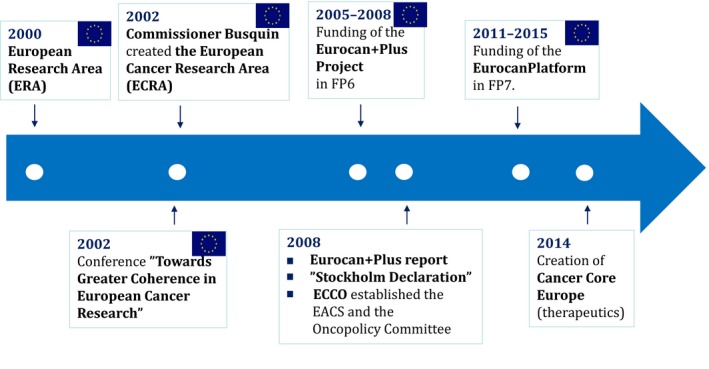
Key events leading to the creation of Cancer Core Europe.

The virtual ECI should combine the best of each nation's initiatives, coordinating efforts and resources towards the primary goal of improving patients’ life and care, and innovating at the forefront of the whole cancer continuum. Such a pan‐European cancer infrastructure would facilitate the integration of efforts geared towards predetermined priorities in prevention, early detection, therapeutics and outcomes research. Sharing and consolidation of patients’ clinical data would not only benefit all research in the area but would provide a platform from which any partnering country can develop their clinical research (trials) using cohorts of patients spanning across Europe. It would also act as a channel for industrial partnerships, offering information and clinical trials unparalleled with anything currently available. Such a virtual ECI could provide an ecosystem where the three strategic priorities put forward by Commissioner Moedas – Open Innovation, Open Science and Open to the World (http://europa.eu/rapid/press-release_SPEECH-15-5243_de.htm) – are interlinked and where the social dimension of science becomes evident. Reaching the final goal, however, will be challenging as illustrated by the considerable time it took to the cancer community and policy makers to establish Cancer Core Europe (Fig. 2), a potential precursor of such a virtual ECI. Cancer Core Europe focuses on therapeutics and aims at addressing the cancer research–cancer care continuum in partnership with six major European cancer centres, most of them CCCs (Celis and Pavalkis, 2017; Celis and Ringborg, 2014; Eggermont *et al*., 2014).

## 2 A mission for cancer 2030: long‐term survival of three of four cancer patients

### 2.1 Cancer prevention

If present knowledge would be used for effective prevention programmes, an incidence reduction of 30–40% may ultimately be expected. The effects of primary prevention are, however, medium to long term and only a moderate impact would be envisaged by 2030. Effects of secondary and tertiary prevention would, together with treatment improvements, increase the 10‐year relative survival rate (taking genders and age into consideration). By 2030, there will also be more information regarding risk factors, premalignant disease as a target for medical prevention and improved strategies for the screening of frequent tumour diseases.

### 2.2 Therapeutics

Structured biomarker research for the identification of patients with microscopic metastases and prediction of therapeutic effects will further support adjuvant systemic treatment and the development of personalized treatment; immunotherapy has an exciting potential regarding long‐term survival; combining anticancer drugs to target tumour driving molecular pathways will increase the therapeutic effect; combination of treatment modalities – radiation therapy and immunotherapy – may further increase treatment effects. Improved diagnostic technologies for early detection and early treatment of high incidence cancers will in the future have an important impact on the cure rate.

Thus, a number of innovative strategies will support the ongoing decrease in mortality due to cancer as well as increase the relative 10‐year survival, an indicator of cure. The 10‐year survival is expected to increase to 75% in European countries with well‐developed healthcare systems, equal to ‘cure of 3 of 4 cancer patients’. The prevention and treatment strategies will pave the way to handling the inequalities in countries with less‐developed healthcare systems.

Cancer Core Europe is presently the only and best response to accomplish the mission. The stepwise process will call for sustainability of its core structure, a gradual inclusion of other CCCs of excellence across Europe, enhanced participation of less‐equipped Member States, as well as coordination with prevention initiatives to create a cohesive scheme for prevention, early detection and treatment.

Below, we provide a brief description of the steps leading to the creation of Cancer Europe and highlight its achievements and actions towards achieving the mission.

## 3 The road map to Cancer Core Europe

Following the creation of the European Research Area (ERA) in the year 2000 (http://www.consilium.europa.eu/en/uedocs/cms_data/docs/pressdata/en/ec/00100-r1.en0.htm) (Fig. 2), which placed science in a central position for the development of a European knowledge‐based economy and society, the cancer community organized itself to proactively advise Commissioner Philippe Busquin regarding future cancer priorities. As a result of these activities and support from the European Parliament (EP), which was a staunch supporter of initiatives to improve cancer research, Commissioner Busquin established the European Cancer Research Area (ECRA) in September 2002 (Fig. 2). In the same year, the European Commission (EC) together with the EP organized a conference in Brussels entitled ‘Towards greater coherence in European Cancer Research’ to identify why Europe was not delivering the outcomes expected by healthcare professionals and patients; cancer research was fragmented, and there was a need to create a common European strategy for cancer research (Celis and Pavalkis, 2017; Celis *et al*., 2013) (http://www.europa.eu/rapid/press-release_SPEECH-02-408_en.htm).

The conference was attended by all key stakeholders, and the Sixth Framework Programme (FP6) that had just started, provided an ideal instrument for making Europe stronger in cancer research (Celis and Pavalkis, 2017; Celis and Ringborg, 2014; Celis *et al*., 2013). For the first time, it was possible for a FP to support clinical research directly. Prompted by the outcome of the meeting and following consultations with the cancer community (Celis and Ringborg, 2014; Celis *et al*., 2013; Saul, 2008), the EC launched a call for proposals in FP6 in 2004 that led to the funding of the Eurocan+Plus project in October 2005, headed by P. Boyle, at that time Director of the IARC (Fig. 2). The consortium was asked to address issues of coordination of cancer research activities in Europe and identify how improved coordination could be implemented through already existing support schemes such as ERA‐NET schemes and Article 169 of the Treaty establishing the European Community (http://www.eurosfaire.prd.fr/7pc/doc/1253547916_06_wolfgang_wittketoolbox.pdf). One of the main recommendations of the Eurocan+Plus project in 2008 was the creation of a platform for translational cancer research, composed of interlinked cancer centres with shared infrastructures and collaborative projects, to facilitate rapid advances in knowledge and their translation into better cancer care (http://ecancer.org/journal/2/full/84-eurocan-plus-report-feasibilitystudy-for-coordination-of-national-cancer-research%20activities.php). Moreover, the report recommended the creation of an ERA‐NET to finance cancer research. A European cancer centre was discussed and agreed upon, provided it would be a virtual structure, and the role of the CCCs was considered critical given the complex infrastructures needed for advanced translational cancer research (Celis and Pavalkis, 2017; Celis and Ringborg, 2014) (http://ecancer.org/journal/2/full/84-eurocan-plus-report-feasibilitystudy-for-coordination-of-national-cancer-research%20activities.php).

**Figure 3:**
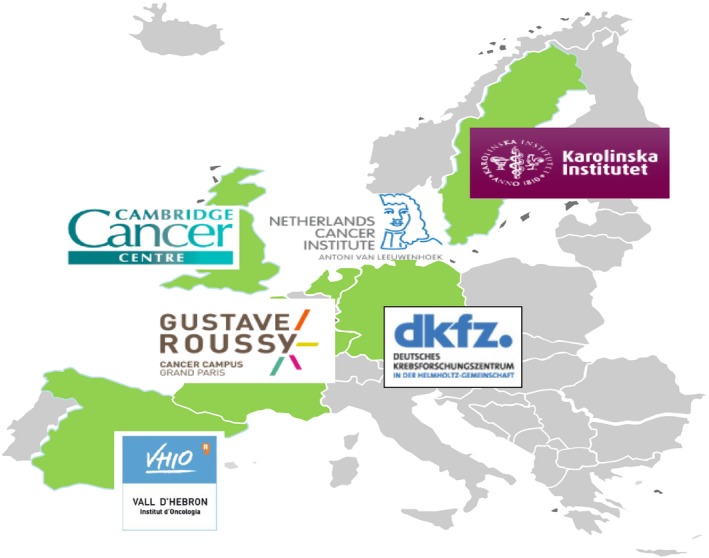
Cancer Core Europe partners. Kindly provided by A. Eggermont, Gustave Roussy.

In 2008, FP6 had ended, and since FP7 had already started in 2007 (with a strong focus on personalized cancer care) (Van de Loo *et al*., 2012), a gap was left that needed to be bridged if the Platform proposal was to become a reality. At this point, the directors of 16 leading European cancer centres met in Stockholm to define the platform concept and to confirm their commitment to its realization they signed the ‘Stockholm Declaration’ (Fig. 2) openly stating their intention to join forces and share resources (Celis and Pavalkis, 2017; Celis and Ringborg, 2014; Celis *et al*., 2013; Ringborg, 2008). Immediately after, at a meeting at the UNESCO headquarters in Paris in 2008 sponsored by the Danish Cancer Society, the Initiative for Science in Europe (ISE), and UNESCO, the first steps towards moving the ‘Stockholm Declaration’ into reality, were discussed with various stakeholders, including former Commissioner P. Busquin (Celis and Pavalkis, 2017; Celis and Ringborg, 2014; Brown, 2009). At this point, it became necessary to engage cancer organizations like the European Cancer Organization (ECCO; Fig. 2) (http://www.ecco-org.eu/), its Policy Committee and advisors, as well as charities like the Danish Cancer Society, and large international organizations such as UNESCO to sustain and accelerate the process (Celis and Pavalkis, 2017; Celis and Ringborg, 2014; Celis and Gago, 2014). The result of these efforts led to the funding of the EurocanPlatform Network of Excellence in FP7 in 2011 (Celis and Pavalkis, 2017; Celis and Ringborg, 2014) (http://eurocanplatform.eu/) (Fig. 2), directed by Ulrik Ringborg from the Karolinska Institutet in Sweden. Twenty‐three cancer centres and five organizations (ECCO, the Organization of European Cancer Institute (OECI), the European Organization for Research and Treatment of Cancer (EORTC), the European Cancer Patient Coalition (ECPC) and eCancer.eu) participated in the project. A main outcome of the consortium was the creation of Cancer Core Europe in 2014 (Fig. 3) (Celis and Pavalkis, 2017; Celis and Ringborg, 2014; Eggermont *et al*., 2014), nearly 12 years after the creation of ECRA by Commissioner Busquin.

## 4 Cancer Core Europe: building an ecosystem for innovation

Cancer Core Europe is a patient‐centred infrastructure that aims at addressing the cancer research–cancer care continuum in partnership with six major European cancer centres (Gustave Roussy Cancer Campus Grand Paris, the Cambridge Cancer Centre, the Karolinska Institutet, the Netherlands Cancer Institute, the Vall D'Hebron Institute of Oncology and the German Cancer Research Centre with its CCC, the National Centre for Tumour Diseases) (Fig. 3; http://www.cancercoreeurope.eu/index.php). Cancer Core Europe was established thanks to a bottom‐up initiative led by A. Eggermont and O. Wiestler triggered by the fact that it was not possible within the EurocanPlatform Network of Excellence project to find appropriate funding instruments that could be implemented within a reasonable short period of time. The leaders of the six centres were faithful to the ‘Stockholm Declaration’ principles and used their own resources to convert a diffuse idea into an object of desire for policy makers and industry; they also provided a breath of hope for patients across Europe.

**Figure 4:**
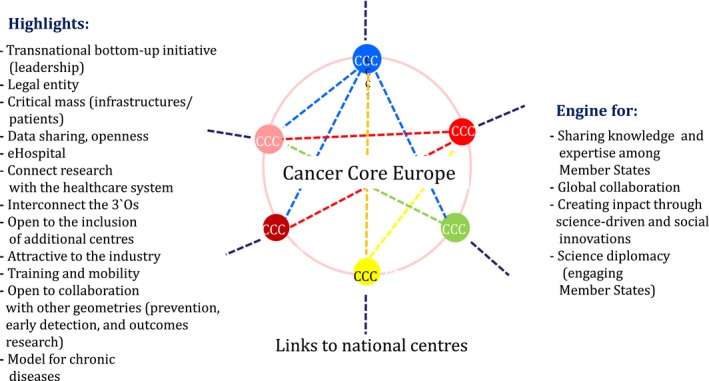
Cancer Core Europe, a hub and an engine to coordinate and optimize European efforts to fight cancer in partnership.

Currently, Cancer Core Europe has significant critical mass; per year, it sees more than 60 000 newly diagnosed patients, treats about 250 000 patients, has more than 1 million consultations and runs about 1500 clinical trials (http://www.cancercoreeurope.eu/index.php). By and large, Cancer Core Europe offers a unique ecosystem where the 3 O's strategic priorities from Commissioner Moedas (http://europa.eu/rapid/press-release_SPEECH-15-5243_de.htm), including science diplomacy, could develop and thrive and it provides a systemic approach to research and innovation (science‐driven and social) as well as international collaboration, facts that may prove decisive for demonstrating the impact of research on society as a whole. Highlights stressing the added value of Cancer Core Europe are summarized in Fig. 4. Being a legal entity, Cancer Core Europe will serve as a hub and an engine to coordinate and optimize joint cancer research efforts across Europe (Fig. 4; http://www.cancercoreeurope.eu/index.php).

### 4.1 Areas of action

The initial aim of Cancer Core Europe, with full data sharing capability, is to focus on the development of new diagnostics and therapeutics to carry out joint translational and clinical research, to develop personalized cancer medicine, to conduct next‐generation clinical trials and to perform outcomes research (Celis and Pavalkis, 2017; Celis and Ringborg, 2014) (http://www.cancercoreeurope.eu/index.php). It has one portal system that greatly facilitates interactions with industry (biotech, high tech and pharma) by speeding up innovative initiatives to reach clinical testing and clinical application.

Carrying out joint translational and clinical research requires the creation of a virtual single e‐hospital, an efficient translational research platform, intercompatible clinical molecular profiling laboratories with a robust computational biology pipeline, standardized functional and molecular imaging, agreed SOP's for molecular diagnostics, ‘omics’, functional genetics and immune‐monitoring, a culture of data collection and storage that allows effective data sharing and outcomes research, as well as excellent basic research, the engine that drives innovation. Cancer Core Europe has already established six coordinated thematic Task Forces in each centre with the aim of pushing cancer research into the next era; these teams address issues such as data sharing, genomics, imaging, clinical trials, immunotherapy, as well as training and education (http://www.cancercoreeurope.eu/index.php).

1. Data Sharing: The aim is to enable patient data sharing (including clinical, pathological and diagnostic imaging, biological and genomic data) in compliance with the local regulations in each member country and to develop tools allowing clinicians to be able to identify individual patients responding to molecular‐targeted therapies. Tools will be put into clinical practice for clinical utility assessment using real‐world patient data to improve treatment decision‐making for individual patients. Gustave Roussy and DKFZ‐NCT have a co‐innovation project in this domain with SAP for data structuring and data normalization. Gustave Roussy has obtained a 1.5 million Euros grant from a charity foundation to develop this programme further and roll it out to Cancer Core Europe with funding for each centre. The consortium has also received a 2 million Euros grant from EIT in 2016 for data sharing and biomarker development.
2. Genomics: A common molecular diagnostics platform will be established. Patient tumour samples will be tested using a standard panel of genes, and the test results will rely upon conventional pipelines for alignment, analysis and variant calling; standard controls will ensure that genomic data are of high quality and uniform. This requires the development and sharing of techniques for sequencing and bioinformatics analysis. Ultimately, these molecular diagnostic methodologies will be used to define clinical groups to determine appropriate therapies. Moving from genes to tumour driving molecular pathways is an important goal.
3. Imaging: This task force will develop companion imaging in the clinical setting allowing healthcare providers to be able to identify individual patients responding to immune therapies, and those who are at risk of developing resistance and relapsing. A partnership in the field of imaging (MRI, PET and CT) will be developed for both quantitative and qualitative analyses, particularly focusing on response to immunomodulatory treatments. Standardization of imaging is not only a prerequisite for a homogeneous set of data, but also necessary to ensure that platforms are uniformly optimized to this highest standard of operation across the participating centres.
4. Clinical trials: The goal is to run new investigator‐initiated clinical trials. These trials will benefit from expediting contracting as well as by the ability to quickly open trials across all members’ centres. Harmonization of the procedures is expected to facilitate interactions with pharmaceutical industry partners. The available expertise in clinical biostatistics and methodology will support innovative next‐generation clinical trial design. At present, a major clinical trial, the Basket of Baskets trial (BOB), is in an advanced stage of planning and is expected to start in the fall of 2017. This trial has various modules around driver pathway targets as well as immunotherapy targets with substantial interest and support from Pharma of more than 12 million Euros. The flexibility of the BOB trial design allows for expansion in cohorts in a ‘rolling on by amendments model’ to speed up the overall new treatments development model. Moreover, in the BOB trial type, further modules of new molecular or epigenetic or immunotherapeutic targets can be added with specific support, to create a continuous innovative clinical trial model (Fig. 5).

**Figure 5:**
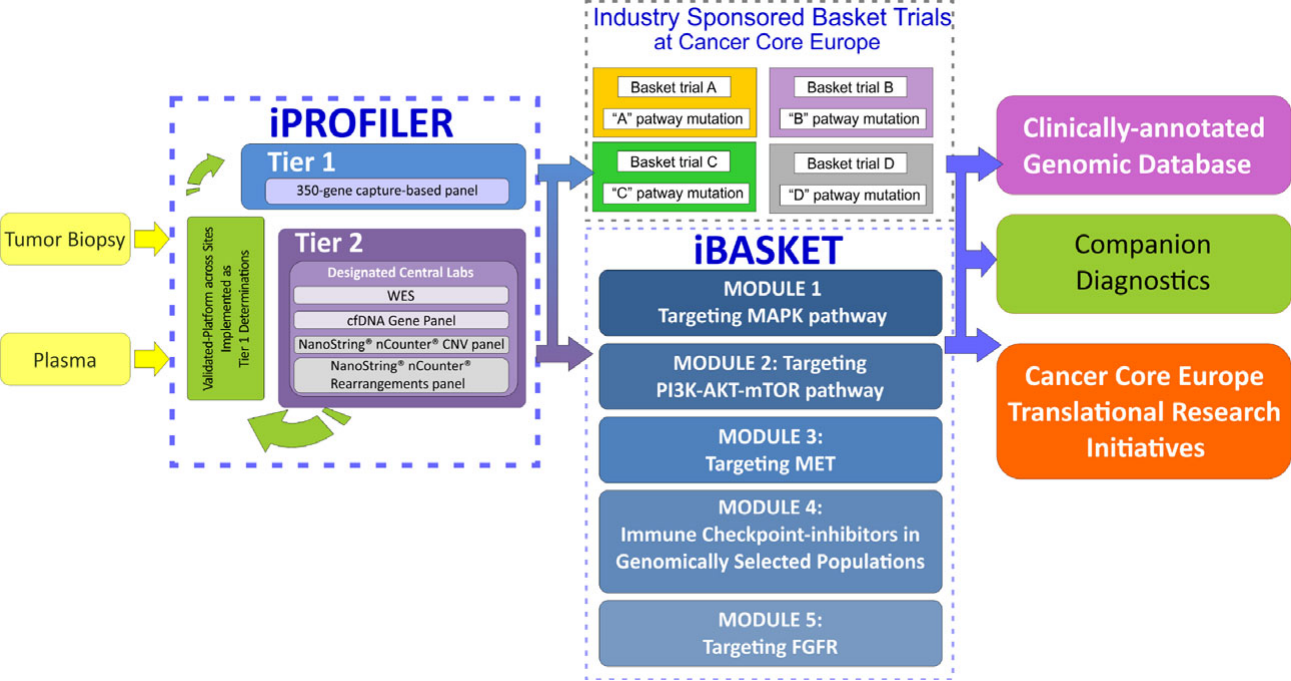
Basket of Basket trial study design. Kindly provided by A. Eggermont, Gustave Roussy.
5. Immunotherapy: Tools will be developed that allow clinicians to identify individual patients that will respond to immunotherapy and those who are at risk of developing resistance and relapsing. Apart from the immunotherapy questions addressed in the BOB core trial, the SABR trial regarding the interaction of radiotherapy in metastatic patients with oligometastatic disease and anti‐PD1‐based immunotherapy was launched in 2017. Neoepitopes generated by genomic alterations in tumour cells will be characterized as well as the TcR responses in the context of various immunotherapeutic approaches, in particular in relationship to modulators such as immune checkpoint blockers and agonists and in relationship to immunogenic cell death agents such as selected chemotherapeutics, targeted drugs and radiotherapeutic approaches.
6. Training and Education: The goal is to develop a common training programme for the benefit of not only the Cancer Core Europe members but also for the broader European scientific and medical community. The education activities will include a Summer School and short‐term courses covering specific topics. Recently, Cancer Core Europe took over the highly successful EurocanPlatform Summer School on Translational Cancer Research, which is held annually in Portugal during October. This year, the Summer School focused on Molecular Cancer Biology and Therapeutics and was attended by more than 100 researchers and clinicians from across Europe. Besides providing a platform for education and dissemination, the school offers excellent networking possibilities. To broaden the expertise, however, it will be necessary to seek collaboration with other basic and clinical organizations such as EACR, OECI, EMBO, FEBS, ESMO and others.

### 4.2 A joint effort – connecting national centres

Cancer Core Europe was exclusive at the start to become inclusive in the long term as a strong pillar was necessary to build and ensure the long‐term success of the initiative. In due course, however, other European CCCs are expected to join Cancer Core Europe based on their capacity to demonstrate scientific excellence. Towards this aim, the EurocanPlatform consortium in collaboration with the EACS has developed quality criteria and methodologies for the designation of CCCs of excellence, which define in particular the level of integrative research to clinical application (Rajan *et al*., 2016). The method was already tested in practice during autumn 2014 and has been in use since the beginning of 2017. In each country, the best CCC will be identified as a candidate for the gradual and controlled expansion of Cancer Core Europe, and the centre is expected to function as the central node within the country (Fig. 4). For example, through programmes like DKTK in Germany, the Specialized Centers programme of CRUK and the SIRIC programme of INCA in France, it will be possible to expand the networks to other centres and hospitals within their countries. Internal connections among the centres, however, are the responsibility of the individual Member States.

Recently, the Istituto Nazionale Dei Tumori di Milano indicated its intention of applying for joining Cancer Core Europe at the beginning of 2018, thus opening the door to a controlled expansion. The inclusion of this CCC will significantly increase the critical mass of the infrastructure.

Some Member States, however, are less equipped than others, and as a result, Cancer Core Europe will operate like an infrastructure – very much like the EMBL and other intergovernmental organizations – to ensure that every country has a role and benefits from this collaborative European endeavour. Cancer Core Europe will facilitate the flow of knowledge and will foster the mobility and training of researchers and clinicians across Europe for short as well as prolonged periods of time. The latter is essential to secure training across disciplines and technologies as well as for building strong networks for future collaboration. The infrastructure will be instrumental in raising capacities in lesser‐equipped countries, and the governance structure will contribute to organizing their resources and activities by actively engaging in science diplomacy.

Cancer Core Europe will also collaborate with centres and individual groups on other continents having shared expertise within its priorities. Targets are the ‘Cancer Moonshot’ initiative in the USA (http://www.nature.com/news/obama-proposes-cancer-moonshotin-state-of-theunion-address-1.19155) and selected large enterprises in China.

## 5 Combining therapeutic and prevention strategies: a cohesive scheme for prevention, early detection and treatment

The creation of Cancer Core Europe had a significant impact on researchers within the area of prevention that recently established a similar initiative – Cancer Prevention Europe – another deliverable of the EurocanPlatform project, led by Christopher Wild, director of the IARC (http://eurocanplatform.eu/news/8267-european-cancer-centresfinally-united-in-long-term-collaborative-partnerships.php). Cancer Prevention Europe brings together European centres of excellence in prevention research and currently include IARC, the Danish Cancer Society, Karolinska Institutet, Stirling University (supported by Cancer Research UK), German Cancer Research Centre, Imperial College London, Wageningen University (supported by World Cancer Research Fund International), European Institute of Oncology and the UK Translational Cancer Prevention Network. The aim of this new consortium is to foster coordination, excellence in research and training alongside dissemination of current best practice in cancer prevention.

Cancer Prevention Europe will be broad in scope covering a spectrum of research from behavioural science to development of novel medical preventative agents. Assessment of the cost‐effectiveness of different interventions, in relation to costs of treatment, care and productivity loss, will be a core component of the initiative. Primary (reducing exposure to risk factors), secondary (detection and treatment at an early stage to stop progression to advanced disease) and tertiary (preventing cancer recurrence) prevention will be covered and emphasis will also be placed on the research evaluation and advocacy dimensions of the prevention agenda.

The agenda for Cancer Prevention Europe would include (i) research into optimizing the implementation of known preventive strategies, (ii) dissemination and research translation to inform policy and practice and (iii) the identification of novel targets for prevention. Specific research activities could include the following areas: cancer registration; cancer aetiology (including recurrence); development and evaluation of preventive interventions; health economics and implementation research to enhance the effectiveness of intervention programmes. These tasks would be supported by a range of platforms, networks and infrastructures and draw together a wide network of partners. Training and capacity building would be integral to the initiative.

The conduct of prevention research and the collation of information on cancer prevention would be translated into an evidence base from which national governments could derive cancer control policy.

To date, Cancer Prevention Europe partners have all contributed financially to the consortium by signing Letters of Agreement in the first instance, thus allowing the creation of the Secretariat at IARC. A formal Consortium Agreement is currently in the process of being finalized, and a position paper reflecting the aims and views of all partners is in its final stage of preparation.

**Figure 6:**
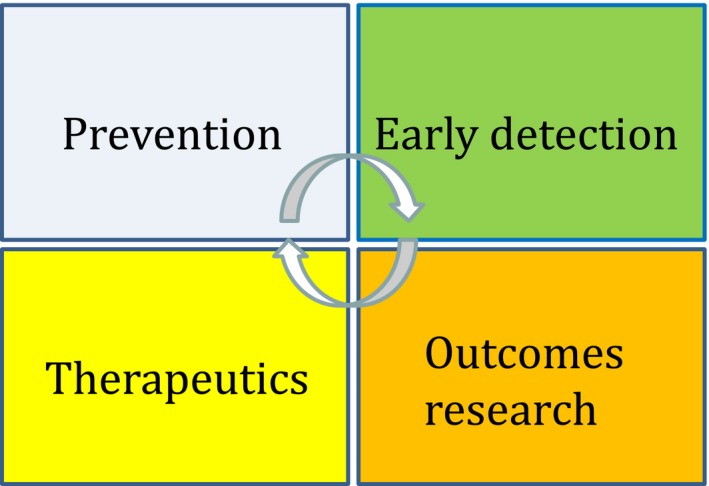
Integrating other research geometries to address the whole cancer continuum. Kindly provided by U. Ringborg, Karolinska Institutet.

Close collaboration between Cancer Core Europe and Cancer Prevention Europe will ensure that developments in cancer biology will translate both in clinical and in population settings. Joining both initiatives (Fig. 6) represents a significant step forward towards the creation in the long term of a virtual ECI that addresses the whole cancer continuum in partnership.

## 6 Can the current EU‐level funding and partnership instruments help?

The success of the virtual infrastructure will very much depend on achieving sustainability over a prolonged period**.** Currently, available EC instruments to support medium long‐term coordination include Article 185, Joint Programming (JPI) and the European Programme co‐fund (EJP co‐fund). Establishing an Article 185 initiative involves a rather lengthy and complicated process that requires long‐term financial commitment from the Member States, a Strategic Research Agenda (SRA), as well as co‐decision with the EP and Council. Setting up a JPI is also a complex and lengthy process as it also requires the firm financial commitment from the Member States and the elaboration of a shared SRA. Moreover, the initiative should address a global challenge involving regional, national and European stakeholders. EJP co‐fund, on the other hand, is simpler in its implementation, and even though issues such as Member States commitment, inclusiveness and others persist, it may represent a practical way forward as an explorative phase is possible through a bridging Coordination and Support Action (CSA) in H2020.

To speed up the process, Cancer Core Europe is currently in the course of identifying ‘champions’ at the highest level among the corresponding Member States. The aim is to work with them and with the Commission to outline a methodology that eventually could pave the way towards sustainability of the infrastructure. Relevant policy issues will be discussed at the first ‘Gago Conference’ tentatively entitled ‘Policy Perspectives for Cancer Research in Europe’ that will take place in Porto, Portugal, in 2018 with the support of Manuel Heitor, Portugal's Minister of Science, Technology and Education. Also, a similar conference jointly organized by the Vatican Pontifical Academy of Science and the EACS is planned to take place at the Vatican City late in 2018.

Accomplishing the mission will also require an increase in the level of financing for basic research, adjusting existing funding mechanisms to boost translational research, as well as the use of structural funds to support less‐equipped Member States.

1. *Increase in the budget allocated to basic research*: The European Research Council (ERC) (https://erc.europa.eu/) is currently the most prestigious funding instrument within the FPs and has contributed considerably to a better understanding of the molecular mechanisms underlying cancer. Given that fundamental research of excellence is the engine driving innovation (Fig. 1), it will be crucial to significantly raise the level of funding allocated to the Agency.
2. *Adjusting existing funding mechanisms to boost translational research*: Adjusting existing funding mechanisms to enhance translational research is also a priority action as current instruments are not fit for the purpose (Celis and Pavalkis, 2017). For example, collaborative research schemes funded by the Framework Programmes (FPs), which have been very fruitful and oversubscribed, do not allow continuation of projects once their funding period terminates, a fact that is incompatible with the extended period it usually takes to translate discoveries into applications. At present, research is carried out under the umbrella of the Innovation Union (http://ec.europa.eu/research/innovation-union/index_en.cfm), and therefore, every effort should be made to ensure the sustainability of successful actions that could have an impact on society. Moreover, there must be a balance between top‐down and bottom‐up approaches to profit from already ongoing collaborative initiatives (Celis and Pavalkis, 2017).

The ERC could also consider the possibility of funding translational research as basic research and innovation are interlinked (Celis and Pavalkis, 2017). The programme could support small groups of independent‐career researchers and clinicians as well as more senior multidisciplinary teams of moderate size. Engaging early‐career investigators in translational research is essential for developing personalized/precision cancer medicine (Celis and Pavalkis, 2017). A further option would be the creation of a ‘European Council for Health Research’ to boost clinical and translational research with a focus on the main chronic diseases. The proposal was initially put forward by the European Alliance for Biomedical Research (BioMed ‘Alliance) in 2012 (Celis and Pavalkis, 2017; Celis and Gago, 2014) (https://www.escardio.org/static_file/Escardio/EU-Affairs/health-research-concept-paper.pdf) and was endorsed by the European Medical Research Councils (EMRC), which proposed the setting up of a ‘European Clinical Research Fund’ ‘working with a bottom‐up approach’ (https://sciencebusiness.net/news/75940/Why-we-need-a-new-strategy-for-health-research-in-Europe), and the European Federation of Pharmaceutical Industries and Associations (EFPIA) (https://www.efpia.eu/news-events/the-efpia-view/statements-press-releases/111206-efpia-fully-behind-the-idea-of-a-european-council-for-health-research/). Recently, the Scientific Panel for Health (SPH) in H2020 at its meeting on 30 June 2017 reinvigorated the proposal after several months of discussions (https://ec.europa.eu/programmes/horizon2020/en/h2020-section/scientific-panel-health-sph) and agreed to prepare a plan of action for political implementation by the end of September this year.

In June 2015, Commissioner Moedas announced his desire to create a European Innovation Council (EIC) to deal with the needs of entrepreneurs and business by supporting company up‐scaling and market creating innovations (http://europa.eu/rapid/press-release_SPEECH-15-5243_en.htm, http://ec.europa.eu/research/eic/index.cfm). More recently, a high‐level group was appointed to advise the Commissioner on this new methodology to boost innovation (http://ec.europa.eu/research/eic/index.cfm). From the health research perspective, however, it is clear that the Council will not support early translational research, and as a result, other funding instruments as the above described must be pursued.

1. *Use of structural funds to support less‐equipped Member States:* A goal of the infrastructure is to ensure that every Member State plays a role and benefits from this collaborative endeavour which is expected to act as a hub for spreading research excellence and for innovation at the EU level under the umbrella of Open Science. By functioning as an infrastructure, it will be possible to ease the flow of knowledge, foster the mobility and training of researchers and clinicians and facilitate the promotion of fruitful collaborations and networking.

To facilitate the participation of lesser‐equipped countries, however, it will be essential to put into operation coordination/funding actions to promote capacity building, training and mobility, access to technologies and instrumentation and equally important changes in the remuneration rules in order to attract the best talents. Achieving this will require a stronger coherence between FP9 and Member States agendas and regulations of the funding mechanisms. Involving structural funds could be carried out under the ‘Spreading excellence and widening participation’ actions, in particular ‘Teaming’ that is geared to support networking of excellence (http://ec.europa.eu/programmes/horizon2020/en/h2020-section/spreading-excellence-and-widening-participation). Another possibility is to establish a new coherent funding mechanism between the EC and the Member States to fit the exact purpose of the mission.

## 7 Governance structure and priorities

Cancer Core Europe and Cancer Prevention Europe have statutes with governance structures; however, the final collaborative governance of the virtual infrastructure will be defined in due course contingent on the type of coordination instrument to be used to sustain the partnership.

Currently, a top priority of the joint structure is to consolidate the research and clinical activities embraced by the various Cancer Core Europe Task Forces (see above) as well as structuring issues concerning the interaction/integration with Cancer Prevention Europe. Further issues include the engagement of other CCCs, interactions with the Member States, regulatory issues, collaborations with industries and business, enhancing patients’ and citizens’ engagement, monitoring progress towards the mission, as well as communication and dissemination.

1. *Engagement of other CCCs:* As mentioned earlier, the development of European criteria for accreditation of CCCs as well as for the designation of CCCs of Excellence are science policy questions that fall under the realm of the EACS. Continuous updating of the Certification standards will be necessary due to constant developments, and therefore, collaboration with the EACS and existing accreditation bodies is essential to stimulate the setting up of new CCCs that are potential candidates for inclusion.
2. *Interactions with the Member States:* There is considerable interest in implementing science diplomacy to engage less‐equipped Member States to participate actively in the joint venture. The aim is to improve training and mobility as well as to encourage them to organize national activities and resources to create a CCC that could connect with additional hospital‐linked regional networks; in this way, it will be possible for them to connect to the hub.
3. *Regulatory issues:* Ethical review processes at the European level will need to be harmonized and simplified to facilitate the translational process and international collaborations. The EACs will provide scientific advice for new regulatory measures to ensure progress and will encourage the contribution of other science and technology fields to stimulate cross‐talk and innovation. Also, criteria must be crafted to monitor research integrity in international research collaborations.
4. *Collaborations with industries and business:* As stated earlier, this is already happening in the case of Cancer Core Europe, and collaborations are expected to flourish as the consortium achieves further visibility due to progressive increase in critical mass and public understanding of its mission.
5. *Enhancing patients’ and citizens’ engagement:* Engaging patients and citizens is central as they must have a clear understanding of the benefits that research and integrative research ecosystems bring to society. The consortium will establish links with large cancer patient organizations and national cancer societies; the aim is to engage them, empower them and voice their needs.
6. *Monitoring progress towards the mission:* Progress will be monitored by having regular meetings of the Executive Committee, annual meetings between Cancer Core Europe and Cancer Prevention Europe, annual reports, input from a Scientific Advisory board (SAB) and possibly a Council.
7. *Communication and dissemination:* The consortium will structure communication to disseminate information to cancer patient organizations, national cancer societies, the cancer research community, policy makers and the press.

## 8 Concluding remarks

By combining innovative prevention and treatment strategies in a sustainable state‐of‐the‐art virtual European cancer centre/infrastructure, it will be possible to achieve a long‐term survival of three of four cancer patients in countries with well‐developed healthcare systems. Furthermore, the concerted actions will pave the way to handling the economic and social inequalities in countries with less‐developed systems. These efforts will also ensure that in the long run, science‐driven and social innovations reach patients across the healthcare systems in Europe.

In the long term, moving towards a virtual ECI will require further harmonization of EU and national priorities and policies, improved research coordination at national, regional and EU level, more efficient and flexible funding mechanisms to provide sustainability, as well as fine‐tuned legal and ethical standards that facilitate international translational research collaborations and the implementation of new diagnostics, therapeutics and preventing technologies. Furthermore, there is a need to create an open Forum where representatives of all the stakeholders can put forward and discuss their ideas and requirements continuously and proactively (Celis *et al*., 2013).

Lastly, the cancer community must continue engaging in science policy activities and inform decision‐makers and civil society at large of the benefits that international collaborative research will bring to both well‐being and national economies.
